# Deciphering the Effect of Lysine Acetylation on the Misfolding and Aggregation of Human Tau Fragment ^171^IPAKTPPAPK^180^ Using Molecular Dynamic Simulation and the Markov State Model

**DOI:** 10.3390/ijms23052399

**Published:** 2022-02-22

**Authors:** Syed Jawad Ali Shah, Haiyang Zhong, Qianqian Zhang, Huanxiang Liu

**Affiliations:** 1 School of Pharmacy, Lanzhou University, Lanzhou 730000, China; syed2017@lzu.edu.cn (S.J.A.S.); zhangqq19@lzu.edu.cn (Q.Z.); 2 Centre for Advanced Drug Research, Abbottabad Campus, COMSATS University Islamabad, Abbottabad 22060, Pakistan; 3 Department of Chemistry, Lanzhou University, Lanzhou 730000, China; zhonghy14@lzu.edu.cn; 4 School of Applied Science, Macao Polytechnic Institute, Macao SAR, China

**Keywords:** Tau, MD simulations, post-translational modifications, acetylation, aggregation, Markov state model

## Abstract

The formation of neurofibrillary tangles (NFT) with β-sheet-rich structure caused by abnormal aggregation of misfolded microtubule-associated protein Tau is a hallmark of tauopathies, including Alzheimer’s Disease. It has been reported that acetylation, especially K174 located in the proline-rich region, can largely promote Tau aggregation. So far, the mechanism of the abnormal acetylation of Tau that affects its misfolding and aggregation is still unclear. Therefore, revealing the effect of acetylation on Tau aggregation could help elucidate the pathogenic mechanism of tauopathies. In this study, molecular dynamics simulation combined with multiple computational analytical methods were performed to reveal the effect of K174 acetylation on the spontaneous aggregation of Tau peptide ^171^IPAKTPPAPK^180^, and the dimerization mechanism as an early stage of the spontaneous aggregation was further specifically analyzed by Markov state model (MSM) analysis. The results showed that both the actual acetylation and the mutation mimicking the acetylated state at K174 induced the aggregation of the studied Tau fragment; however, the effect of actual acetylation on the aggregation was more pronounced. In addition, acetylated K174 plays a major contributing role in forming and stabilizing the antiparallel β-sheet dimer by forming several hydrogen bonds and side chain van der Waals interactions with residues I171, P172, A173 and T175 of the corresponding chain. In brief, this study uncovered the underlying mechanism of Tau peptide aggregation in response to the lysine K174 acetylation, which can deepen our understanding on the pathogenesis of tauopathies.

## 1. Introduction

The formation of β-sheet-rich neurofibrillary tangles (NFT) caused by the abnormal aggregation of misfolded microtubule-associated protein Tau is a hallmark of tauopathies such as Alzheimer’s Disease (AD), frontotemporal dementia with Parkinsonism (FTDP-17), Progressive Supranuclear Palsy (PSP), Pick’s disease (PD) and others. The aggregation of such toxic species in the human brain has been established previously to positively correlate with the progression of cognitive decline in such patients and is used as a marker of Braak’s staging [1,2,3,4]. Under normal physiological conditions, Tau stabilizes the microtubules ensuring cytoskeletal organization and cellular trafficking [5]. Meanwhile, post translational modifications (PTMs) such as phosphorylation, acetylation, methylation, ubiquitination and several others play a key role in modulating the function of Tau proteins. In AD brain, Tau protein undergoes abnormal post-translational modifications such as hyperphosphorylation [5] and enhanced acetylation [6]. Although abnormal phosphorylation and acetylation have an effect on the accumulation of tau, many studies [7,8] strongly suggested that it is the acetylation rather than the phosphorylation of human Tau peptides that leads to the aggregation of recombinant Tau in vitro [9].

Possible sites of lysine acetylation in Tau reported previously mainly include K163, K174, and K180 [6,10]. There are also some other potential sites of possible acetylation including residue K274, K280, K281 and K369 [11]. Recently, Min et al. [10] found that the acetylation of Tau at K174 (ac-K174) is the critical pathological change affecting Tau homeostasis and toxicity in early AD brain by means of in vitro and in vivo study of tau-acetylation using different mutations mimicking the acetylated (K174Q mutant) and the de-acetyl mimicking state (K174R mutant) [10]. However, it is still unclear why the acetylation of Tau at K174 increased the toxicity of Tau protein. Given that the origin of Tau toxicity is the misfolding and abnormal aggregation, will the acetylation of Tau at K174 affect Tau misfolding and aggregation? Uncovering the acetylation effects on the structural feature and aggregation propensity of Tau will be valuable in understanding the origin of Tau-induced toxicity due to the acetylation at the specific position K174 and the pathogenic mechanism of Tauopathies.

However, it is extremely challenging to reveal the effect of acetylation on Tau structure by conventional experimental approaches. On the one hand, it is difficult to obtain proteins acetylated at specific sites by conventional experimental methods. On the other hand, experimental techniques such as spectroscopic techniques, x-rays diffraction, circular dichroism and immunobiology can only provide macroscopic phenomena, and it is difficult to determine the mechanism of Tau aggregation at the molecular level [10,11,12,13]. Compared with the experimental method, molecular dynamics simulation can give a detailed description of the dynamic process of protein folding-misfolding and aggregation [14,15,16], and it is easy to obtain post-translational modified Tau proteins at specified sites. Therefore, molecular dynamics simulation methods are especially suitable for studying the effect of post-translational modifications on the conformational features of Tau protein, and the correlated studies have been previously reported [17,18].

In this study, to disclose how acetylation of K174 affects the aggregation of Tau protein and investigate the effect of actual acetylation and amino acid mutation mimicking acetylation, several microseconds MD simulations were carried out to study the spontaneous aggregation of Tau protein with actual acetylation and mutation mimicking acetylation of K174 by using Tau decapeptide (^171^IPAKTPPAPK^180^) as a model. The formation of dimers is the first step of aggregation. Thus, uncovering the dimerization mechanism is the key point in understanding the aggregation process. Here, the conformational transition of monomers into dimers under the influence of acetylation is determined first. Following Markov State Model (MSM) analysis [19], the mechanism of initial nucleation was uncovered and the critical transition states were identified.

## 2. Results and Discussion

### 2.1. Acetylation and Mutation of Lysine Residue (K174) Affects the Overall Structure of Tau Fragment ^171^IPAKTPPAPK^180^ Monomer

Initial structures of the fragments were obtained using the tleap utility of Amber 18 [20]. Lysine residue (K174) of the decapeptide ^171^IPAKTPPAPK^180^ was mutated to glutamate mimicking acetylated lysine (KQ) and arginine mimicking the de-acetylated lysine residue (KR) [10]. The actual acetylation of the lysine (ac-K174) residue was carried out using FF_PTM [21,22].

**Figure 1:**
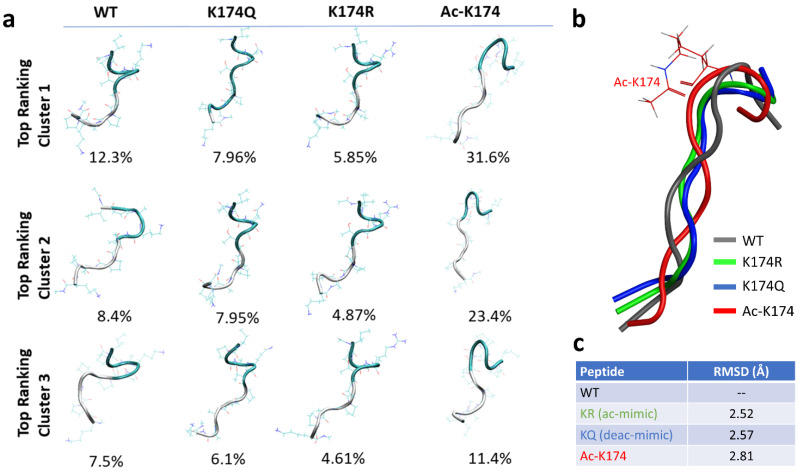
Initial structure of the differently modified ^171^IPAKTPPAPK^180^ Tau fragments obtained by Replica Exchange Molecular Dynamics. (**a**) Cluster analysis of the top-most highly populated clusters. (**b**) Superimposed structures of differently modified Tau fragments. (**c**) Root mean square deviation of differently modified Tau fragments. The WT, ac-K174, KQ and KR is shown in grey, red, blue, and green color, respectively.

To obtain more reliable initial states of the modified and non-modified peptide fragments, Replica Exchange Molecular Dynamics (REMD) were run first [23]. Eight replicas of each system at a temperature range of 270–560 K were run for 200 ns and the conformations were collected at 300 K. Then, the conformations were clustered using K-means clustering, as shown in Figure 1a. The highest-ranking conformation from each top-ranked cluster (Figure 1b) was then selected for subsequent studies. The Root Mean Square Deviation (RMSD) of each selected fragment was then calculated against the native wild type (WT)—non-modified K174 containing monomers. As shown in Figure 1c, the highest deviation of 2.81 Å was found for the ac-K174 containing monomers followed by the glutamate mutated (KQ) ac-mimic and arginine mutated (KR) deacetyl mimic containing mutated monomers. The higher deviation in terms of RMSD of acetylated fragments suggested that the modification of K174 affected the overall structure of the individual peptide fragment.

### 2.2. Spontaneous Aggregation of Acetylated (ac-K174) and Acetylation Mimic (ac-Mimic KQ) Containing Peptides

To investigate the effect of K174 acetylation on Tau aggregation and whether the effect of actual acetylation and mimic-acetylation have similar effects on Tau aggregation, four spontaneous aggregation systems were constructed, including native WT, ac-K174, ac-mimic KQ and deac-mimic KR, each system including 16 monomers. For each system, the monomer was selected from the top ranked clusters, 16 monomers were randomly placed in a cubic box with minimum distance of around 10 Å between each monomer, and then 2 µs molecular dynamics simulations were performed in explicit solvent environment. Prior to production runs, the simulation systems have been equilibrated to around 300 K and 1 atmospheric pressure. Production runs of 2 µs were then performed on the equilibrated simulation systems. The RMSD monitoring during the simulation process, as shown in Figure S1, proves that most of the simulations are up to equilibration after 1 µs, indicating that our selection simulation time scale is reasonable.

**Figure 2:**
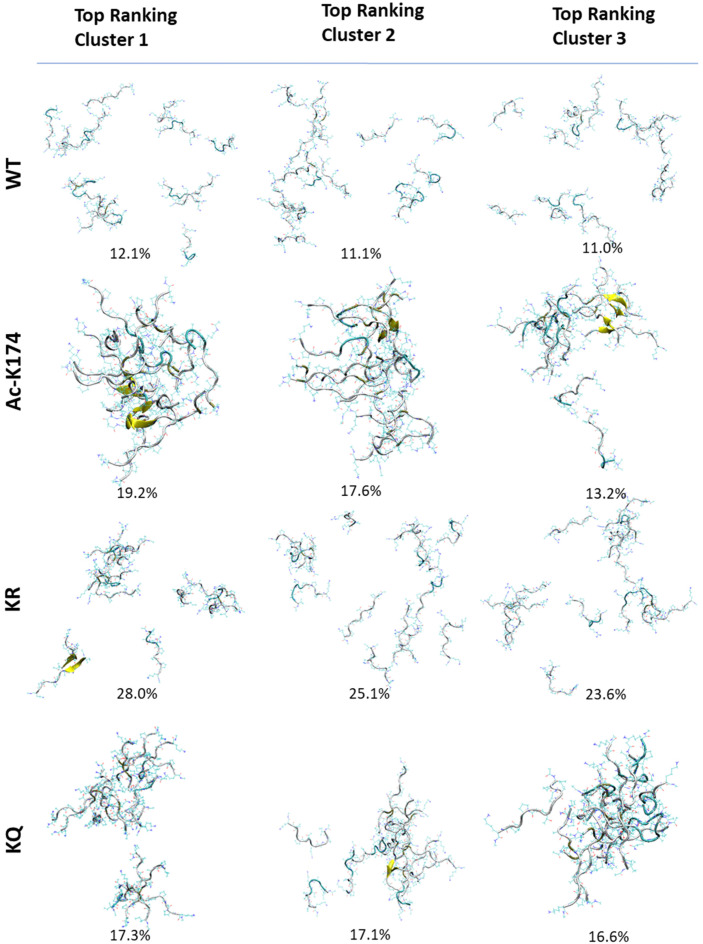
Cluster analysis of differently modified ^171^IPAKTPPAPK^180^ Tau fragments. Top ranking three clusters from each modified Tau 16-chain oligomers are depicted.

To investigate the overall structural changes during the spontaneous aggregation of different systems, cluster analysis based on K-means algorithm was performed and the results are given in Figure 2 and Figure S2, respectively. Firstly, cluster analysis was performed to identify the most prominent conformations of 16-chain oligomers obtained by MD simulation. As shown in Figure 2, in the case of native WT 16-chain oligomers, the cluster analysis showed that that there is no obvious difference between each cluster. In all clusters of the native WT system, the peptides tend to move far apart from each other, and the aggregation tendency is low. In the case of the ac-K174 16-chain oligomers, reversely, there was an obvious aggregation tendency and the formation of β-sheet structure in the top three clusters (up to 50% in total). In the case of ac-mimic KQ 16-chain oligomers, initially the peptides were scattered far apart from each other. As the simulation proceeded further, they tended to move towards each other and form more associations and aggregates. There is also a marked increase in the β-sheet propensity and dimerization. While in the case of the deac-mimic KR system, even in the highly populated clusters there are many peptides that do not make any kind of association with other peptides. Other highly populated clusters have similar distribution with that of the native WT 16-chain oligomers.

Furthermore, the cluster population of Tau fragment 16-chain oligomers with respect to time elapsed is shown in Figures S2 and S3. Cluster analysis was performed using K-means clustering based on their cartesian coordinates and the analyses were carried out with a 1 ns time interval. As shown in Figure S2a, the ac-K174 16-chain oligomers tend to associate and aggregate immediately. As the simulation proceeded, they remained associated with each other and there is minute change in overall secondary structure. In contrast, the native WT 16-chain oligomers do not aggregate with each other. With elapsing time, the peptide fragments tend to move far apart from each other (Figure S3a). Additionally, the ac-mimic KQ 16-chain oligomers tend to aggregate from 500–700 ns (Figure S2b). With elapsing time, the peptides remain associated with each other with a much smaller fraction of disassociation. As shown in Figure S3b, for the deac-mimic KR 16-chain oligomers, initially the peptides seemed to have some degree of association with each other. However, the association is not consistent throughout the simulation period.

The above analyses indicated that the 16-chain oligomers of ac-K174 exhibited immediate association and aggregation as compared to that of the non-acetylated WT 16-chain oligomers, which signifies the effect of acetylation on the aggregation of Tau fragments. In the case of ac-mimic KQ mutated 16-chain oligomers, initially, negligible aggregation was observed. However, as the simulation proceeded the fragments started to aggregate like the ac-K174 16-chain oligomers. This proves the hypothesis that acetylation or mutations mimicking the acetylated state has a positive effect on the overall aggregation of the Tau peptide fragments. On the other hand, the deac-mimic KR 16-chain oligomers were observed to show a much similar pattern to that of the native WT 16-chain oligomers.

**Figure 3:**
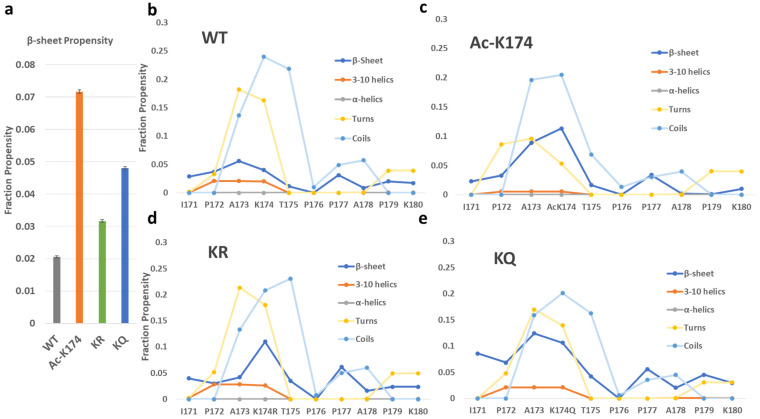
Average secondary structure profile of differently modified ^171^IPAKTPPAPK^180^ Tau fragments as predicted by the DSSP analysis. (**a**) The total β-sheet propensity of differently modified ^171^IPAKTPPAPK^180^ Tau fragments. (**b**–**e**) The per-residue secondary structure profile of differently modified ^171^IPAKTPPAPK^180^ Tau fragments.

To reveal the structural changes during the simulation, the secondary structure analysis based on the whole trajectory and the evolution of β-sheet structure of each residue over a time period of 2 μs were calculated by DSSP [24] analysis, and the obtained results were shown in Figure 3 and Figure S4. The secondary structure profile of the acetylated and mutated fragments varies from that of the non-modified fragments. As shown in Figure 3 and Figure S4, the ac-K174 16-chain oligomers have the highest β-sheets propensity compared with other systems. The highest difference in the β-sheet propensity can be seen among the WT and ac-K174 16-chain oligomers. The β-sheet propensity of the glutamate mutated ac-mimic KQ 16-chain oligomers remained slightly higher than that of the deac-mimic KR 16-chain oligomers and native WT 16-chain oligomers system. The deac-mimic KR 16-chain oligomers have a very similar pattern to that of the WT 16-chain oligomers, which is shown in Figure S4. This indicates that, to a certain extent, actual acetylation and mimic-acetylation have slightly different effects on the structure of Tau protein, which could be attributed to the shorter side chain and the absence of an amino group in glutamine mutated 16-chain oligomers compared with that of the acetylated lysine containing 16-chain oligomers. As shown in Figure 3b–e, secondary structure analysis of the peptides shows a considerably larger percentage of turn and coils. Change in the secondary structure can be solely associated with the type of modification occurring at the lysine residue. It is noteworthy that the acetylation (ac-K174) or ac-mimic mutations (K174Q) at K174 position also tend to improve the β-sheet propensity of the adjacent residues as shown in Figure 3b–e. Due to the fact that acetylation or acetyl-mimic mutation increases in the β-sheet propensity of the Tau oligomers, there is a decrease in the turns and coils propensity of the ac-K174 and KQ 16-chain oligomers. A higher β-sheet propensity of Tau fragments in aggregates has been advocated by previous studies [10], and our findings also suggest that acetylation (ac-K174) or acetylation-mimicking mutations (KQ) tend to change the overall secondary structure from random turns and coils to enhanced β-sheet propensity.

**Figure 4:**
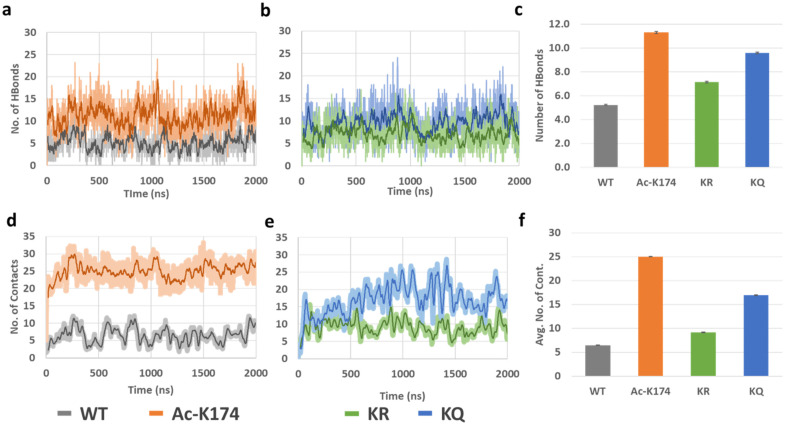
(**a**–**c**) Hydrogen bonds and (**d**–**f**) Interpeptide contacts of differently modified 171IPAKTPPAPK180 Tau 16-chain oligomers during the entire MD ensemble.

To further investigate the mechanism by which K174 acetylation affects Tau aggregation, time evolution of the inter-chain backbone hydrogen bonding and contacts of all the systems were calculated and the corresponding results were shown in Figure 4. As shown in Figure 4a–c, the ac-K174 16-chain oligomers have been observed to form the largest number of hydrogen bonds between peptide chains resulting in more compact aggregates, whereas that of the native WT 16-chain oligomers showed very few inter-peptide hydrogen bonds. On average, the total number of inter chains hydrogen bonds of all fragments fluctuated at around 10~15 in the case of ac-K174 16-chain oligomers, whereas that of native WT 16-chain oligomers contained around only five hydrogen bonds on average. The ac-mimic KQ 16-chain oligomers also showed higher number of hydrogen bonds compared with that of the non-modified WT and deac-mimic KR 16-chain oligomers.

Interpeptide contact analysis displayed a very similar pattern to that of the hydrogen bond analysis, as shown in Figure 4d–f. The average number of contacts were plotted to determine any visible difference (Figure 4f). The acetylated (ac-K174) and ac-mimic KQ 16-chain oligomers were observed to form the highest number of inter-peptide contacts in contrast to that of non-modified WT and deac-mimic KR 16-chain oligomers. The average number of interpeptide contacts formed by the ac-K174 16-chain oligomers were fluctuating at around 15~25 versus 5~10 in the case of the non-modified native WT 16-chain oligomers. In the case of the ac-mimic KQ versus deac-mimic KR 16-chain oligomers, a similar pattern was found.

**Figure 5:**
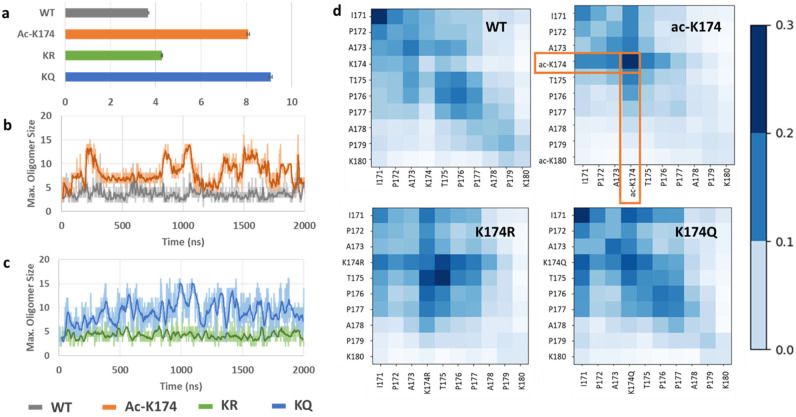
Oligomerization of differently modified 16-chain oligomers of ^171^IPAKTPPAPK^180^. (**a**) Average number of oligomers formed by differently modified Tau 16-chain oligomers during the entire MD ensemble, (**b**) comparison of maximum oligomer size of ac-K174 vs native WT 16-chain oligomers, (**c**) comparison of maximum oligomer size of ac-mimic KQ vs deac-mimic KR 16-chain oligomers, and (**d**) Inter-residue contact map of differently modified Tau 16-chain oligomers.

Furthermore, to investigate the oligomerization effect of different modifications, the maximum oligomer size of the respective peptides was analyzed. As shown in Figure 5a, the ac-K174 16-chain oligomers were observed to exhibit the maximum oligomerization state with the average number of peptides spanning around 8–10. The acetylation mimicking KQ mutated 16-chain oligomers have an average number of oligomer size around 9–12. The native WT 16-chain oligomers and the de-acetylated mimicking KR 16-chain oligomers demonstrated the least oligomerization with respect to time. Furthermore, to identify key residues during the spontaneous aggregation, inter-residue contact maps were constructed [25] and the results were shown in Figure 5d. The contact map displays the probability of residues contact during oligomerization. For the ac-K174 16-chain oligomers system, the acetylated lysine ac-K174 depicts the highest number of inter-residue contacts. Followed by the acK174–acK174 contacts, the highest number of contacts was found for I171–acK174, P172–acK174, A173–acK174 and T175–acK174, respectively. Similar to the ac-K174 system, the KQ mutated peptides were shown to have a higher number of intrapeptide contacts. The notable contacts include I171-I171, K174Q–K174Q, I171–K174Q and T175–K174Q, respectively. For the non-modified WT peptides, a higher number of intrachain I171–I171 interactions were prominent. From Figure 5, due to the acetylation of K174 in ac-K174 16 chains, the ac-K174-ac-K174 contacts occur frequently and thus the I171–I171 number of contacts remains lower, whereas due to the absence of the acetylated K174, there are no ac-K174-ac-K174 contacts and therefore the I171-I171 contacts are of the highest number for both KQ and WT 16 chains. In the case of deac-mimic KR, the highest number of intrapeptide interactions of T175–T175 and K174R–T175 was observed. Thus, from the interpeptide contact analysis it is evident that the acetylation of K174 or its ac-mimic mutation K174Q renders the monomers to associate with each other more frequently and high order oligomers are thus formed.

### 2.3. Dimerization Mechanism of the Tau ^171^IPAKTPPAPK^180^ Fragment under the Influence of Lysine Acetylation (ac-K174)—Insights from MSM Analysis

**Figure 6:**
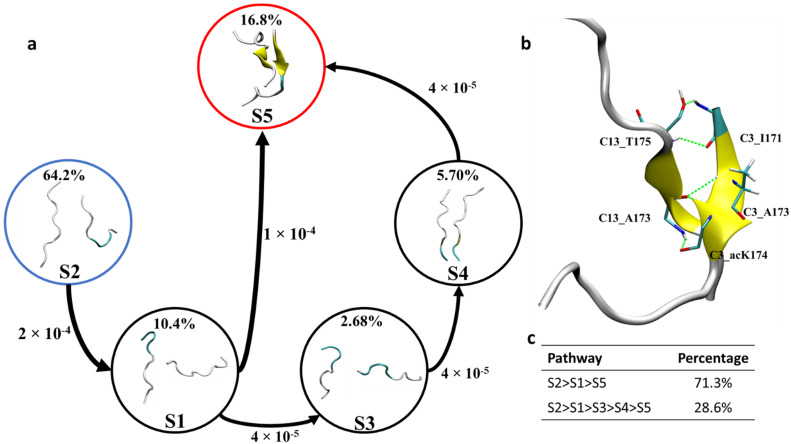
(**a**) Representative macrostates identified by MSM and network transition pathway of ac-K174 dimers in spontaneous aggregation. (**b**) C3–C13 dimer formed during the 2 µs MD ensemble and the interpeptide hydrogen bonds (**c**) percentage of each transition pathway. The dominated coarse-grained fluxes are shown with an arrow size corresponding to the transition rate. The percentage abundance of each macrostates is given in percentages.

The formation of dimers is the first step of aggregation. Therefore, uncovering the formation mechanism of dimers is of utmost importance in understanding the initial aggregation process. In the ^171^IPA(ac-)KTPPAPK^180^ system, C3–C13 dimers can form stable dimers enriched with β-sheet structure from the cluster analysis of 2 µs MD trajectories. The dimer possesses antiparallel conformation. To uncover the formation mechanism of this dimer here, Markov state model (MSM) analysis was performed based on the 2 µs long MD trajectories. To determine the Markovian behavior of the constructed MSM, an implied time scale was plotted as a function of lag time. The implied time scale versus the lag time for C3–C13 dimer is given in Figure S5. As shown in Figure S5a, the implied time scale curves reached a constant beyond 10 ns for the dimer. Therefore, a lag time of 10 ns was used to construct the MSM. The convergence of the implied time scales does not necessarily imply that the MSM is Markovian. Thus, the constructed MSM is further validated by the Chapman-Kolmogorov test (C-K test) [26]. As shown in Figure S5b, when the 300 microstates were lumped into five macrostates, the estimated curves fit well with the predicated curves, indicating that the model has Markovian properties. Therefore, based on the C-K test, the microstates were lumped in to five macrostates by the Robust Perron cluster analysis PCCA+ method. The representative conformations are shown in Figure 6 [27]. The macrostate S2 similar to the initial non-aggregating monomers served as a starting point for the transition pathway. The macrostates S5 identified by the MSM accounted for about 16.8% of the conformational space compared with 2.68%, 5.7% and 10.4% for macrostates S3, S4 and S1, respectively. The highly populated macrostates S5 with extended β-sheet conformations were used as the target state.

As shown in Figure 6, transition pathway analysis of C3–C13 dimers revealed that the most abundant extended antiparallel β-sheet dimer, i.e., macrostate S5 can be formed via two different pathways. Firstly, the initial macrostate S2 transitions to macrostate S5 via S1. This pathway is the dominant transition pathway and encompasses about 71.3% of the total transitions. The second pathway (28.6% of the total transition) is the transitioning of macrostate S2 into macrostate S5 via macrostates S1, S3 and S4, in turn.

**Figure 7:**
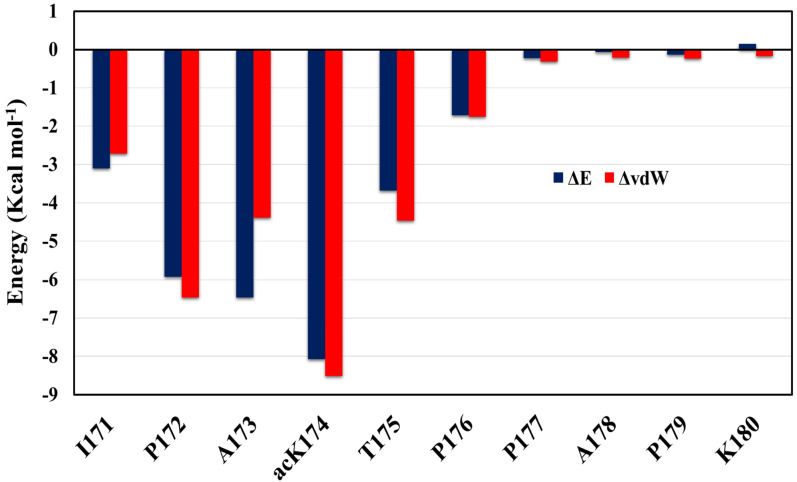
Per-residue energy contribution of acK174 (C3–C13) dimer. The difference in total energy and van der Waal’s energy is shown in the blue bar graph and orange line graph, respectively.

To identify key interactions and residues responsible for the formation of the C3–C13 dimer, hydrogen bond analysis, residue interaction network (RIN) and energy analyses were performed for the most populated state with extended β-sheets, and obtained results are shown in Figure 6b and Figure 7. As shown in Figure 6b, for macrostate S5 of the antiparallel dimer C3–C13, the residue C3:I171 was found to form two hydrogen bonds with C13:T175, each residue serves to provide a hydrogen bond donor and an acceptor, respectively. The two hydrogen bonds formed during the MD ensemble were found to have percentage occupancy of about 11.20% and 23.10%, respectively. The residue A173 of chain C3 and C13 was also found to stabilize the antiparallel dimer by forming hydrogen bonding interaction between C3:A173@NH and C13:A173@O. The percentage occupancy for intrachain A173 hydrogen bonding interaction was found to be 31.05%. The most notable hydrogen bonding interaction that stabilizes the acetylated Tau dimer is the formation of hydrogen bonding interaction between C3:acK174 and C13:A173. A hydrogen bond donor and an acceptor from each residue was found to stabilize the β-sheet dimer formation. The percentage occupancy of each hydrogen bond formed is about 34.5%. Hydrogen bonds formed by the ac-K174 provide additional stabilization to the dimer formation, which may not be possible in non-acetylated WT peptides and is the underlying reason for enhanced peptides interaction to form dimers and high order oligomers. Details of the hydrogen bonds donor and acceptors stabilizing the β-sheet dimer formation is given in Table 1.

To further identify the driving force of the formation of the dimer, the inter-peptide binding free energy of the dimer identified was determined using MM-GBSA (Molecular Mechanics—Generalized Born Surface Area) calculations [28,29]. Binding free energy of the dimer C3–C13 macrostate S5 identified by the MSM analysis is determined. A total of 200 frames were extracted and analyzed for energy calculations. Difference in energy terms is given in Table 2. It is evident that dimerization of antiparallel C3–C13 dimer was energetically favorable (ΔG_bind_ = −29.2 kcal/mol). The van der Waals interaction and electrostatic interaction also serves as the main driving force for dimer formation. Figure 7 shows total energy contribution and vdW energy contribution ΔvdW. As can be seen in Figure 7, the per-residue decomposition of the binding free energy profile revealed that the acetylation of Tau at K174 has a tremendous effect on dimer stabilization and subsequent aggregation. Followed by the contribution of ac-K174, the residues I171, P172, A173 and T175 also have considerable contribution towards to the dimer stabilization which is possible only due to the acetylation of ac-K174 which forms hydrogen bonding and van der Waal interactions with the adjacent residue and is evident from oligomerization, hydrogen bonding and interpeptide contact analysis.

## 3. Materials and Methods

### 3.1. Simulation Details

The Tau peptide ^171^IPAKTPPAPK^180^ initial structure was constructed and MD simulations were carried out using Amber 18 [20]. The peptide was modified to the ac-mimic by replacing lysine (K174) residue with Glutamate (KQ) and the deac-mimic mutated state by replacing lysine (K174) with arginine (KR). Acetylation of lysine residue (ac-K174) was carried out by using the FF_PTM library [21]. To further refine and obtain more reliable initial structures, Replica Exchange Molecular Dynamic (REMD) simulations [23] at a temperature range of 270–560 K were carried out and the conformations at 300 K were collected for analysis and further use. K-means clustering of the REMD simulation was carried out and the top ranked representations from the top-ranked clusters were selected for subsequent studies. To investigate spontaneous aggregation, a 16-chain oligomers system was constructed by placing peptides randomly at an approximate distance of 10 Å from each other. Here, the letter C was used to represent a ^171^IPAKTPPAPK^180^ monomer in 16-chain oligomers. For example, C1 represents the first ^171^IPAKTPPAPK^180^ monomer in 16-chain oligomers.

Amber 18 with Amber ff99SB force field, which has been successfully applied to a simulation of the folding and aggregation of peptides [20,30,31,32], was used for a molecular dynamics simulation. Each system was immersed in a cubic box of the TIP3P water model [33] neutralized with chloride counter ions to retain electroneutrality of the simulation systems. All systems were minimized using the steepest descent method followed by the conjugate gradient energy minimization method. In the case of the REMD simulation at temperature range of 270–560 K, eight replicas were subjected to an initial canonical (NVT) ensemble followed by the isothermal-isobaric (NPT) ensemble, and a 200 ns production run with the time step set to 2 fs was performed. As for the simulations of 16-chain oligomers, each system was gradually heated from 0 to 300 K in an NVT canonical ensemble with Langevin thermostat, and coupling coefficient of 2.0 ps^−1^ was used. To calculate long-range electrostatic interactions, the Particle Mesh Ewald (PME) method was used and a cut-off distance of 10 Å was set for non-bonded interactions [34]. To restrain bonds involving hydrogen atoms, the SHAKE algorithm was used [35]. The equilibration of the simulation systems and later production runs were carried out in the isothermal isobaric (NPT) ensemble. All systems were then simulated for 2 µs with a time step of 2 fs.

Analysis of the MD simulation was carried out using the CPPTRAJ utility of Amber 18. K-means clustering was used to differentiate the MD simulation data based on Cartesian coordinates. For hydrogen bond analysis, if the distance between donor D and acceptor A was less than 3.5 Å and the angle D-H-A was greater than 150°, a hydrogen bond was considered to be formed. For inter-peptide contacts, if distance between two backbone atoms is less than 4.0 Å then a contact is considered to be formed. DSSP analysis was used to assign a secondary structure profile to the peptides [24]. The oligomerization state and size were determined using python script previously reported [25].

### 3.2. Markov State Model (MSM) and Transition Pathway Analysis

The MSM analyses for dimerization process were carried out by PyEMMA software [19,36]. MSM analysis can partition the conformational space into several metastable macrostates, where each macrostate identified includes the conformations with similar structural and dynamic properties. The backbone Cα distances and the torsion angles ϕ, ψ and ω were used as input co-ordinates for the C3–C13 dimer in the case of all-atom explicit simulations. To reduce the dimensionality and to retain a maximum of 90% total kinetic variance, the time-lagged independent component analysis (TiCA) algorithm was used. Conformations from MD simulations were then clustered into microstates and implied time scales were determined [26,37,38]. The average transition time between two subsets of states is represented by the Implied time scale. A lag time of about 10 ns is applied in the case of all-atom explicit simulations.

Validation of the constructed models was carried out by the Chapman-Kolmogorov test (C-K test) [26,36]. The calculated transition probabilities from simulation data were compared with corresponding transition probabilities from MSM [39]. The microstates were then lumped into macrostates using the determined lag time with the Robust Perron-cluster analysis (PCCA+) method [27].

### 3.3. Binding Free Energy and Per-Residue Energy Contribution in Dimer Formation and Stabilization

The binding free energy and the per-residue energy contribution for the dimers identified were performed using the MM-GBSA method [28,40]. Energy contribution was estimated by calculating the individual species (dimer, monomer1, and monomer2) energy terms, and then the change in binding free energy is calculated by the following formula:

ΔG_Bind_ = G_dimer_ − G_monomer1_ − G_monomer2_. (1)

### 3.4. Residue Interaction Network Analysis

The interchain residue network analysis was used to map the interaction of intra-chain residues. Based on the atomic distance measurements and nature of the contacts formed between the residue pairs, a two-dimensional graph illustrating interaction is plotted. Application of the residue interaction network analysis in protein-ligand binding and protein folding has been reported previously [39,41,42]. Here, we identified the residue interaction network for the most dominant macrostates identified by the MSM analysis with the Ring web server [43]. Visualization and analysis of the interaction network was performed using the Cytoscape Software [44].

## 4. Conclusions

In this study, we investigated the effect of lysine acetylation (ac-K174) on the misfolding and aggregation of the Tau peptide ^171^IPAKTPPAPK^180^ by applying the long-time all-atom molecular dynamics simulation and Markov state model analysis. The obtained results indicate that the acetylation at K174 can increase the aggregation propensity of the studied Tau fragment due to increased inter-chain hydrogen bonding interactions and contacts. In addition, mutation mimicking the acetylated state of the Tau peptide has a similar effect on the aggregation pattern as that of actual acetylation, but not to the same extent. Among the formed oligomer of the ac-K174 16-chains system, the dimer formed between C3 and C13 has antiparallel β-sheets orientation and is relatively stable. Thus, to further analyze the dimerization mechanism, MSM analysis was performed. The dominant transition pathway from separate monomers to antiparallel β-sheet structure was identified from S2 to S5 via S1. The further hydrogen bond analysis and binding free energy calculation indicate that the residue ac-K174 is a key contributing residue involved in the spontaneous aggregation of the peptides by forming several hydrogen bonds and van der Waals interactions with residues I171, P172, A173, T175 and P176. Our study provides the molecular basis of lysine acetylation affecting the aggregation of Tau protein and will deepen the understanding of the function of abnormal post-translational modification on the pathogenesis of tauopathies. The current study about the influences of lysine acetylation on Tau aggregation can also provide valuable ideas and useful insights for exploring the action of other post-translational modification such as methylation or ubiquitination on tauopathies.

## Figures and Tables

**Table 1:** Hydrogen bonds stabilizing the dimer formation and their percentage occupancy during MD ensemble.

Acceptor	Donor	Occupied Percentage (%)
C3-acK174@O	C13-A173@N	34.35
C13-A173@O	C3-acK174@N	34.35
C13-A173@O	C3-A173@N	31.05
C3-I171@O	C13-T175@N	23.10
C13-T175@O	C3-I171@N	11.20
C3-I171@O	C13-T175@N	9.75

**Table 2:** Binding free energy (kcal/mol) of C3–C13 dimers as calculated by MM-GBSA calculations.

System	ΔEvdw	ΔEelc	ΔEGB	ΔEsurf	ΔGgas	ΔGsol	ΔGbind
C3–C13	−31.1	−8.6	14.2	−3.7	−39.7	10.5	−29.2

## Data Availability

Not applicable.

## Supplementary Materials

The following are available online at https://www.mdpi.com/article/10.3390/ijms23052399/s1.
