# Supplementary material for: Deciphering the Effect of Lysine Acetylation on the Misfolding and Aggregation of Human Tau Fragment 171IPAKTPPAPK180 Using Molecular Dynamic Simulation and the Markov State Model
Source: Int J Mol Sci. 2022 Feb 22;23(5):2399. doi: 10.3390/ijms23052399 (PMC8910285; doi:10.3390/ijms23052399)
Supplement: Supplementary file 1 [file ijms-23-02399-s001.zip › ijms-1568122-supplementary.pdf]

Supplementary Materials

Deciphering the Effect of Lysine Acetylation on the Misfolding and Aggregation of Human Tau Fragment ^{171}IPAKTPPAPK^{180} Using Molecular Dynamic Simulation and the Markov State Model

Syed Jawad Ali Shah [1,2,†], Haiyang Zhong [3,†], Qianqian Zhang [1] and Huanxiang Liu [1,4,*]

[1] School of Pharmacy, Lanzhou University, Lanzhou 730000, China; syed2017@lzu.edu.cn (S.J.A.S.); zhangqq19@lzu.edu.cn (Q.Z.)
[2] Centre for Advanced Drug Research, Abbottabad Campus, COMSATS University Islamabad, Abbottabad 22060, Pakistan
[3] Department of Chemistry, Lanzhou University, Lanzhou 730000, China; zhonghy14@lzu.edu.cn
[4] School of Applied Science, Macao Polytechnic Institute, Macao SAR, China
* Correspondence: hxliu@lzu.edu.cn; Tel./Fax: +86-931-891-5686
† These authors contributed equally to this work.

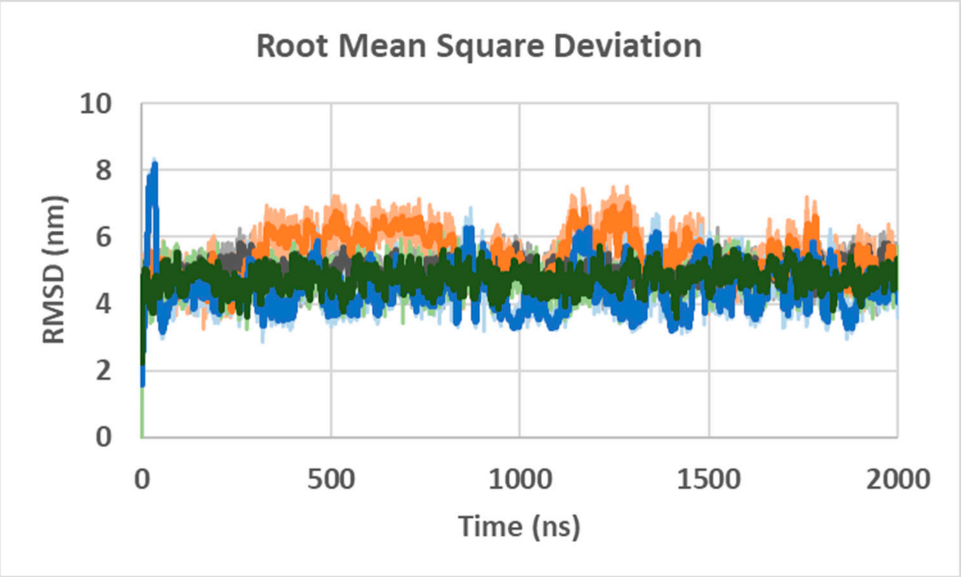

Figure S1. RMSD profile of Differently Modified 16-mer of ^{171}IPAKTPPAPK^{180}. The WT non-modified peptides, acetylated K174, acetyl-mimic KQ peptides and deacetyl-mimic KR is shown in grey, red, blue, and green color respectively.

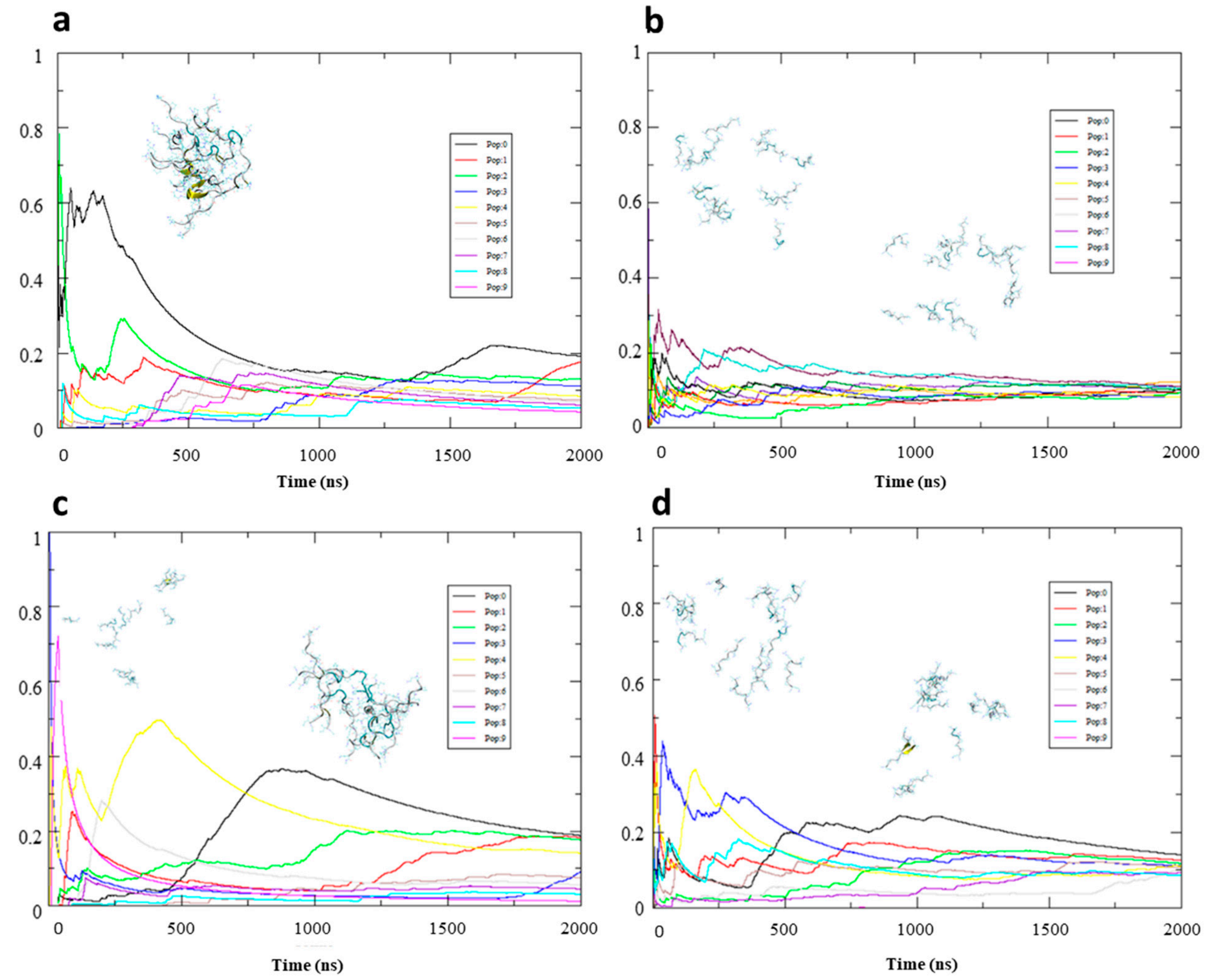

Figure S2. Time-lapse Cluster Analysis of Differently Modified 16-mer of [171]IPAKTPPAPK[180]. (a) acetylated K174 16-mers, (b) non-modified WT 16-mers, (c) The acetyl-mimic KQ mutated 16-mers and (d) the deacetyl-mimic KR mutated 16-mers. Different Cluster populations are shown in different colors.

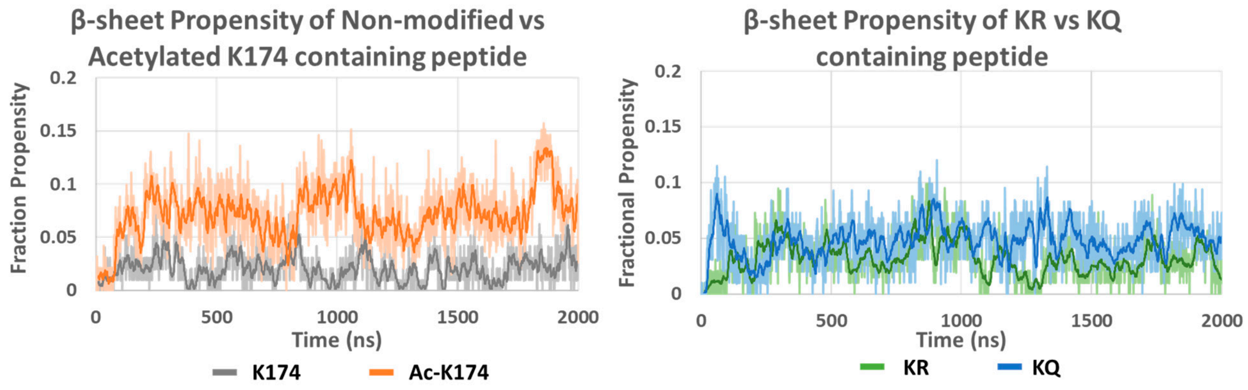

Figure S3. β-sheets propensity of differently modified [171]IPAKTPPAPK[180] tau fragments as predicted by DSSP analysis. The non-modified WT, ac-K174, KQ and KR is shown in grey, orange, blue and green color, respectively.

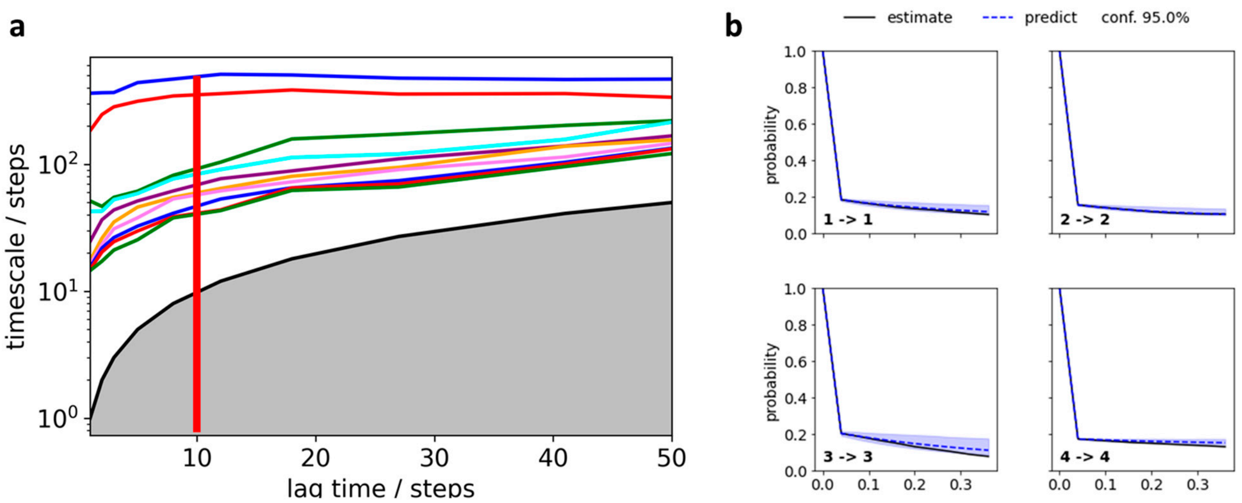

Figure S4. Markov state model validation for C3-C13 Dimer. (a) Implied relaxation timescale as a function of lag time τ= 10 ns (10 steps) is appropriate and (b) Chapman-Kolmogorov test curves obtained by lumping 300 microstates into 5 macrostates.
